# Undernutrition-induced substance metabolism and energy production disorders affected the structure and function of the pituitary gland in a pregnant sheep model

**DOI:** 10.3389/fnut.2023.1251936

**Published:** 2023-11-15

**Authors:** Shuai Liu, Huizhen Lu, Shengyong Mao, Zijun Zhang, Wen Zhu, Jianbo Cheng, Yanfeng Xue

**Affiliations:** ^1^College of Animal Science and Technology, Anhui Agricultural University, Hefei, China; ^2^Biotechnology Center, Anhui Agricultural University, Hefei, China; ^3^College of Animal Science and Technology, Nanjing Agricultural University, Nanjing, China

**Keywords:** substance transport and metabolism, energy production, cytoskeleton, immune response, pituitary

## Abstract

**Introduction:**

Undernutrition spontaneously occurs in ewes during late gestation and the pituitary is an important hinge in the neurohumoral regulatory system. However, little is known about the effect of undernutrition on pituitary metabolism.

**Methods:**

Here, 10 multiparous ewes were restricted to a 30% feeding level during late gestation to establish an undernutrition model while another 10 ewes were fed normally as controls. All the ewes were sacrificed, and pituitary samples were collected to perform transcriptome, metabolome, and quantitative real-time PCR analysis and investigate the metabolic changes.

**Results:**

PCA and PLS-DA of total genes showed that undernutrition changed the total transcriptome profile of the pituitary gland, and 581 differentially expressed genes (DEGs) were identified between the two groups. Clusters of orthologous groups for eukaryotic complete genomes demonstrated that substance transport and metabolism, including lipids, carbohydrates, and amino acids, energy production and conversion, ribosomal structure and biogenesis, and the cytoskeleton were enriched by DEGs. Kyoto encyclopedia of genes and genomes pathway enrichment analysis displayed that the phagosome, intestinal immune network, and oxidative phosphorylation were enriched by DEGs. Further analysis found that undernutrition enhanced the lipid degradation and amino acid transport, repressing lipid synthesis and transport and amino acid degradation of the pituitary gland. Moreover, the general metabolic profiles and metabolic pathways were affected by undernutrition, repressing the 60S, 40S, 28S, and 39S subunits of the ribosomal structure for translation and myosin and actin synthesis for cytoskeleton. Undernutrition was found also to be implicated in the suppression of oxidative phosphorylation for energy production and conversion into a downregulation of genes related to T cell function and the immune response and an upregulation of genes involved in inflammatory reactions enriching phagosomes.

**Discussion:**

This study comprehensively analyses the effect of undernutrition on the pituitary gland in a pregnant sheep model, which provides a foundation for further research into the mechanisms of undernutrition-caused hormone secretion and metabolic disorders.

## 1. Introduction

Pituitary gland is the junction between the brain and the rest of body (1). It receives signals from the brain and secretes a variety of hormones such as the growth hormone, thyroid stimulating hormone, adrenocorticotropic hormone, luteinizing hormone, follicular stimulating hormone, and prolactin, etc. (2). These hormones released from the pituitary gland participate in physiological activities such as metabolism, growth and development, reproduction, and lactation (3). The pituitary gland exhibits a high degree of developmental plasticity throughout life, which means it can continuously alter the proliferation of specific cell types in response to changes seen in the organism or the environmental (4). Significant physiological and anatomical changes occur in the maternal pituitary gland during conception, and these are essential for the normal development of the fetus (5). Therefore, a pituitary gland with normal function is very important to maternal metabolism and pregnancy as well as fetal growth and development. It has been demonstrated that nutritional status (excessive or deficient nutrition) and diet composition affect the synthesis and secretion of hormones in the pituitary gland (6). Nutrition can alter the balance between pituitary follicular stimulating hormone secretion and gonadal feedback (7). Therefore, maternal undernutrition during late gestation may affect both maternal and fetal metabolism and health through influencing the pituitary gland.

Related studies about the pituitary and nutrition mostly focused on the effects of maternal undernutrition on the hypothalamic-pituitary-adrenal axis (8, 9) and hypothalamic-pituitary-gonadal axis (10) in the fetus. A study in sheep has proved that maternal malnutrition can reduce the response of the male fetal pituitary gland to gonadotropin-releasing hormone (11). Prenatal malnutrition reduced the sensitivity of the hypothalamic-pituitary-adrenal axis in offspring rats (12). Some studies showed that the endocrine system of the body could be affected by undernutrition. For instance, undernutrition influenced metabolic, immune, and neuroendocrine functions in female rats (13). Similarly, research has shown that long-term feed restriction in sheep can disrupt pituitary function and lead to neuroendocrine abnormalities (14). Additionally perinatal malnutrition disrupted the neuroendocrine system in ewes (15). However, little attention has been paid to the structure, metabolism, and function of maternal pituitary glands upon undernutrition so far. Therefore, this study aimed to explore the effects of malnutrition during late gestation on the pituitary gland of pregnant ewes and its potential mechanisms.

A hypothesis was proposed that undernutrition of ewes during late gestation might change the expression of genes involved in substance transport and metabolism, energy production and conversion, cell components, and biological processes in maternal pituitary gland and finally affect its and function. In this study, we established an undernutrition model of pregnant ewes to study the influence of undernutrition on maternal pituitary gland based on unbiased transcriptome and metabolome analysis.

## 2. Materials and methods

### 2.1. Animal management and sample collection

The animal experiment was approved by the Institutional Animal Care and Use Committee of Anhui Agricultural University (No. SYDW-P20190600601). Twenty Hu-sheep with similar body conditions (body weight: 53.9 ± 2.9 kg), 108 days of gestation, similar parities (2-3 parities), and multiple lambs (2-4 lambs) were selected as the research objects. All ewes were fed *ad libitum* for a preliminary 7-day period to determine the feed intake (1.74 kg/d total mixed ration, dry matter basis), and they were randomly divided into two groups at 115 days of gestation. Ewes in the CON group (*n* = 10) were fed *ad libitum*, while ewes in the TR group (*n* = 10) were fed 30% of the normal feed intake (0.52 kg/d total mixed ration, dry matter basis) to establish an undernourished model. Each pregnant ewe was fed in a single pen (9:00 and 15:00) and drank water freely. Daily feed intake of each pregnant ewe was recorded daily throughout the experimental period to measure average daily feed intake. Body weight was recorded on the day at the start and end of the experiment. After 15 days of treatment, we slaughtered all the ewes at 130 days of gestation when an undernourished model was established successfully and pituitary tissue and serum samples were collected.

### 2.2. Chemical analysis

Analysis of routine nutritional components in diet were analyzed according to the AOAC protocol (16). Briefly, dry matter content was determined in the oven at 105°C for 24 h until the weight was constant. Crude ash content was determined in a muffle furnace at 550°C for 12 h. Crude protein and ether extract contents were measured using an Automatic Kjeldahl analyzer K9840 (Hanon, Jinan, China) and a Crude Fat analyzer SZF-06A (Xinjia, Shanghai, China), respectively. The neutral detergent fiber and acid detergent fiber contents were determined using the method reported by Van Soest et al. (17). The diet formula and nutrient compositions were detailed in Supplementary Table 1. Serum levels of pituitary hormones were measured using chemiluminescence immunoassay. Growth hormone (GH) was measured with the Siemens IMMULITE 2000XPi chemiluminescent immunoassay analyzer and adrenocorticotropic hormone (ACTH) with the Auto Lumo A2000 Plus fully automated chemiluminescent assay analyzer.

### 2.3. Transcriptome sequencing

Ten pituitary tissue samples were selected randomly from the two groups (five from each group) for transcriptome sequencing assay. Firstly, total RNA was extracted from the frozen pituitary tissue samples using the TRIzol method (Takara Bio, Otsu, Japan). The purity, concentration, and integrity of the RNA samples were tested to ensure the quality of RNA samples used for transcriptome sequencing. Secondly, mRNA enriched from total RNA by the magnetic bead method [magnetic bead with Oligo (dT)] was randomly fragmented by a fragmentation buffer. The first cDNA strand was synthesized using mRNA as a template. Subsequently, buffer solution, dNTPs, RNase H, and DNA polymerase I were added to synthesize the second cDNA strand. Purified cDNA was ligated to sequencing adapters and amplified using the NEBNext Ultra RNA library preparation kit. Fragments of an appropriate length were selected to obtain a cDNA library. The quality of the cDNA library was checked using Bioanalyzer 2100 system (Agilent Technologies, Santa Clara, CA, USA). Finally, the library was sequenced on a Hi-Seq 2500 platform based on the sequencing by synthesis technology (Biomarker Company, Beijing, China).

### 2.4. Transcriptome data analysis

Reads containing adapters and low-quality reads were removed from the raw data to obtain high-quality clean reads, which were mapped to sheep reference 4.0 using HiSAT2 (18) and assembled using String Tie (version 1.3.4D) to calculate the values of fragments per kilobase of exon model per million mapped fragments (FPKM) of all genes (19) to evaluate gene expressional levels. Differentially expressed genes (DEGs) between the CON and TR groups were identified using DESeq2 (version 1.6.3) with the criteria of false discovery rate (FDR) < 0.05 and fold change (FC) > 2 or <0.5 (TR/CON). A biomarker company cloud platform was used for volcano plot, clusters of orthologous groups for eukaryotic complete genomes (KOG) functional taxonomy, and Kyoto encyclopedia of genes and genomes (KEGG) pathway enrichment analysis.

### 2.5. Quantitative real-time PCR

Quantitative real-time PCR (RT-qPCR) was used to detect the expressional levels of genes involved in the transport and metabolism of lipids, carbohydrates, and amino acids, ribosome synthesis, the cytoskeleton, energy production and conversion, and phagosomes in the pituitary gland. Gene primers are detailed in Supplementary Table 2. PCR mixture consisted of a 20 μL reaction system, including 10 μL SYBR Master Mix, 0.8 μL of each primer, 0.4 μL ROX Reference Dye II primer, 2.0 μL cDNA template, and 6 μL nuclease-free water. The thermal cycle conditions were briefly described as follows: denaturation and activation of Taq polymerase at 95? for 30 s followed by 40 thermal cycles of 95? for 5 s and 60? for 34 s. After the amplification process, the melting curve was plotted by heating plate (95? for 15 s, 60? for 60 s, and 95? for 15 s). Fluorescence detection was performed using an Applied Biosystem 7500 (Thermo Fisher Scientific, Waltham, MA, USA). The relative mRNA expression levels of genes were calculated by 2^–Δ^ ^Δ^ *^Ct^* method, and β-actin was used as the housekeeping gene.

### 2.6. Sample preparation for LC/MS detection

Ten pituitary tissue samples were selected randomly from the two groups (five from each group) for metabolome analysis. A 100 mg pituitary tissue sample was homogenized with 1000 μL tissue extracting solution (67.5% methanol, 7.5% chloroform, and 25% H_2_O), which was then centrifuged for 10 min at 4?, 13,700 x *g*. The supernatant was transferred to a new 2 mL centrifuge tube, concentrated, dried, and redissolved in 200 μL 50% acetonitrile solution and 2-chloro-l-phenylalanine (4 mg/L) was used as the interior label. The solution was filtered by 0.22 μm membrane and transferred into a sample bottle for LC-MS detection using a Vanquish UHPLC System (Thermo Fisher Scientific, USA) with ACQUITY UPLC HSS T3 column (150 × 2.1 mm, 1.8 μm) (Waters, Milford, MA, USA) and an Orbitrap Exploris 120 (Thermo Fisher Scientific, USA) with ESI ion source. The temperature of the LC column was maintained at 40?, and the flow rate and injection volume were set at 0.25 mL/min and 2 μL, respectively. For LC-ESI (+)-MS analysis, the mobile phases consisted of (B2) 0.1% formic acid in acetonitrile (v/v) and (A2) 0.1% formic acid in water (v/v). For LC-ESI (-)-MS analysis, the analytes were carried out with (B3) acetonitrile and (A3) ammonium formate (5 mM). The parameters of MS were as follows: sheath gas pressure 30 arb, aux gas flow 10 arb, spray voltage 3.50 kV and −2.50 kV for ESI (+) and ESI (-), respectively, capillary temperature 325?, MS1 resolving power 60,000 FWHM, number of data dependent scans per cycle 4, MS/MS resolving power 15,000 FWHM, and normalized collision energy 30%. The metabolite name was reported if the difference between theoretical mass and observed mass was less than 0.003%. Then the metabolites were obtained by annotating the Human Metabolome Database,^1^ LipidMaps,^2^ and mzClound^3^ database according to the MS/MS fragment mode. Pathway analysis and enrichment analysis of different metabolites were conducted on the MetaboAnalyst 5.0 web server.^4^

### 2.7. Statistical analysis

The independent-sample *t*-test using IBM SPSS Statistics 25 (SPSS Inc., Chicago, IL, USA) was performed to assess the intake, growth performance, serum levels of pituitary hormones, and differences in gene expressional levels between the two groups. Data were shown as means ± standard errors of the means, and *P* < 0.05 indicated significant difference. The principal component analysis (PCA) and the partial least squares discriminant analysis (PLS-DA) of total genes and total metabolites were conducted using SIMCA-P v.14.1 software (Umetrics, Umea, Sweden). DEGs in the transcriptome were selected using the criteria of FDR < 0.05 and FC > 2.0 or <0.5. Different metabolites in metabolome were selected using the criteria of variable importance in projection (VIP) > 1, *P* < 0.05, and FC > 1.5 or < 0.67.

## 3. Results

### 3.1. Growth performance of ewes

The ratio of feed restriction for the TR group was 32.11% compared to the CON group (Table 1), which was extremely close to the designed level 30%. At the start of the experimental period, no significant difference (*P* = 0.590) was observed in the average body weight between the CON and TR groups. After 15-day treatment, the body weight of ewes in the TR group was significantly lower than that in the CON group (*P* < 0.001). The average daily weight gain of the TR group was negative, which was also significantly lower than that in the CON group (*P* < 0.001).

**TABLE 1:** Growth performance and daily feed intake of ewes in the CON and TR groups.

Item	CON	TR	SEM	*P*-value
Initial weight, kg	54.2	53.5	0.939	0.590
Final weight, kg	56.0	44.1	0.906	<0.001
Daily weight gain, g/d	118.0	−624.7	38.755	<0.001
Daily feed intake, kg/d	1.62	0.52	0.004	<0.001

### 3.2. Total genes analysis

**FIGURE 1:**
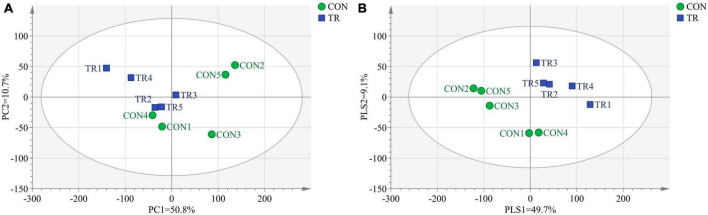
Principal component analysis (PCA) and partial least squares discriminant analysis (PLS-DA) of total genes in pituitary gland for ewes in the control group (CON, normal feeding level, *n* = 5) and treated group (TR, the feeding level is limited to 30%, *n* = 5). **(A)** PCA score scatter plot; **(B)** PLS-DA score scatter plot (predictive ability parameter (Q2) (cum) = 0.362, goodness-of-fit parameter (R2) (Y) = 0.998).

In transcriptome analysis, we got 69.70 Gb clean data for the 10 samples. The GC contents for the 10 samples ranged from 49.83 to 51.22% and the Q30 bases in all the samples were higher than 92.23%, indicating the high quality of these clean data. The mapping ratios of clean reads to the reference genome (Oar v4.0) were between 89.04% and 91.86%, showing the high mapping efficiency. In order to analyze the total transcriptome profiles, we conducted PCA and PLS-DA of total genes in pituitary gland of all the ewes from the CON and TR groups. As shown in Figure 1A, the PCA plot basically separated the two groups with axis 1 and 2 explaining 50.8 and 10.7% of the total variation, respectively. The PLS-DA plot completely separated the two groups, and axis 1 and 2 explained 49.7 and 9.1% of the total variation, respectively (Figure 1B).

### 3.3. Differentially expressed genes selection and analysis

**FIGURE 2:**
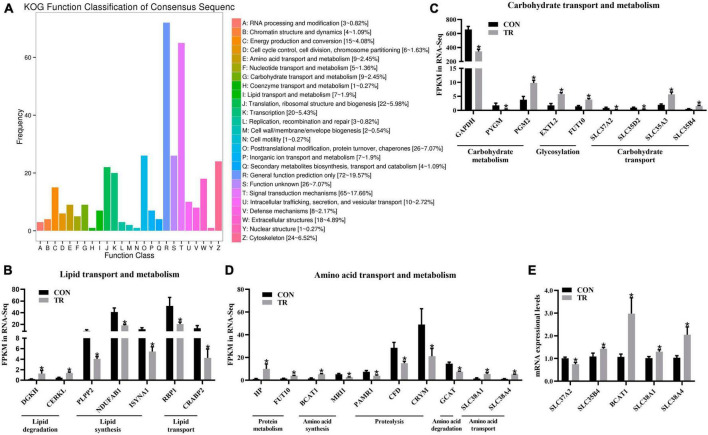
Undernutrition changed substance transport and metabolism in the pituitary gland. **(A)** KOG function classification of DEGs in pituitary gland between the CON and TR groups. The x axis represents KOG function terms, and the y axis represents the number of DEGs. The expression of genes related to lipid transport and metabolism **(B)**, carbohydrate transport and metabolism **(C)**, and amino acid transport and metabolism **(D)** in transcriptome sequencing. **(E)** The expression of genes related to carbohydrate transport, amino acid synthesis, and amino acid transport in quantitative real-time PCR. Data were presented as the mean with SEM. DEGs were selected based on FDR < 0.05 and FC > 2 or <0.5 (*n* = 5 per group). The difference of gene expression in quantitative real-time PCR was identified by independent sample *t*-test (*n* = 10 per group), and asterisk indicated the significant difference (*P* < 0.05).

Based on statistical analysis, a total of 581 DEGs were screened out between the two groups. As shown in Supplementary Figure 1, compared with the CON group, 152 DEGs were upregulated and 429 DEGs were downregulated in the TR group. For further analysis, KOG functional classification of these DEGs in the pituitary gland was performed. As shown in Figure 2A, these terms, including translation, ribosomal structure and biogenesis (J), cytoskeleton (Z), energy production and conversion (C), amino acid transport and metabolism (E), carbohydrate transport and metabolism (G), and lipid transport and metabolism (I), were obviously enriched by DEGs in addition to general function prediction only (R) and signal transduction mechanisms (T). In order to better understand the functional changes of these terms, DEGs enriched in these terms were further analyzed according to the gene ontology annotation.

### 3.4. Undernutrition induced disorders of substance transport and metabolism

For lipid transport and metabolism, DEGs involved in diacylglycerol degradation, i.e., DGKH and CERKL, were both upregulated in the TR group compared with the CON group (Figure 2B). DEGs associated with diacylglycerol synthesis (PLPP2), fatty acid biosynthesis (NDUFAB1), inositol biosynthesis (ISYNA1), retinol transport (RBP1), and retinoic acid transport (CRABP2) were downregulated in the TR group (Figure 2B). For carbohydrate transport and metabolism, DEGs related to carbohydrate catabolism, including GAPDH and PYGM, were downregulated in the TR group, except for PGM2 (Figure 2C). In contrast, DEGs associated with glycosylation and carbohydrate transport, including EXTL2, FUT10, SLC35A3, and SLC35B4, were upregulated, except for SLC37A2 and SLC35D2 (Figure 2C). For amino acid transport and metabolism, DEGs related to protein metabolism and amino acid synthesis, including HP, FUT10, and BCAT1, were all upregulated while MRI1 was downregulated in the TR group compared to the CON group (Figure 2D). All these DEGs related to proteolysis and amino acid degradation, including PAMR1, CFD, CRYM, and GCAT, were downregulated in the TR group compared with the CON group (Figure 2D). In addition, the DEGs involved in amino acid transport, SLC38A1 and SLC38A4, were both upregulated in the TR group compared with the CON group (Figure 2D). Some DEGs involved in substance transport and metabolism were randomly selected for the RT-qPCR assay, and results showed that SLC37A2 (*P* = 0.015) was downregulated while SLC35B4 (*P* = 0.044), BCAT1 (*P* = 0.015), SLC38A1 (*P* = 0.017), and SLC38A4 (*P* = 0.011) were upregulated in the TR group compared with the CON group, which was highly consistent with the results of transcriptome sequencing and confirmed the validation of sequencing data (Figure 2E).

### 3.5. Undernutrition changed metabolic profiles and metabolic pathways in the pituitary gland

**FIGURE 3:**
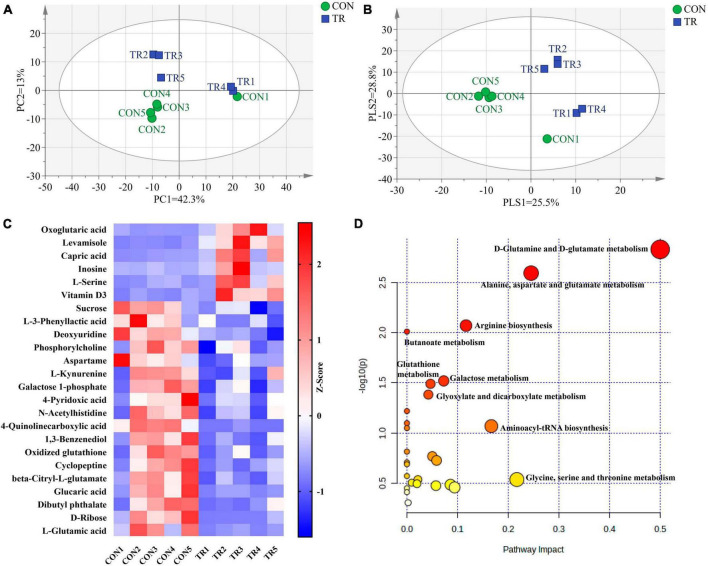
Undernutrition changed metabolic profiles and metabolic pathways of the pituitary gland. Principal component analysis (PCA) and partial least squares discriminant analysis (PLS-DA) of total metabolites in the pituitary gland of ewes in the CON and TR: **(A)** PCA score scatter plot; **(B)** PLS-DA score scatter plot. **(C)** Heatmap of the levels of different metabolites in pituitary gland between the two groups. **(D)** Pathway analysis of different metabolites in pituitary gland.

PCA (Figure 3A) and PLS-DA (Figure 3B) plots of total metabolites demonstrated that the total metabolic profiles of the pituitary gland were changed obviously upon undernutrition during late gestation. In total, 24 different metabolites were identified between the CON and TR groups (Table 2). Compared with the CON group, 6 different kinds of metabolites were increased in the TR group while 18 different kinds of metabolites were decreased. The changes of different metabolites between the CON and TR groups are shown on the heat map (Figure 3C). KEGG pathway enrichment analysis of these different metabolites demonstrated that D-glutamine and D-glutamate metabolism (*P* = 0.001), alanine, aspartate, and glutamate metabolism (*P* = 0.003), arginine biosynthesis (*P* = 0.008), butanoate metabolism (*P* = 0.010), galactose metabolism (*P* = 0.030), glutathione metabolism *(P* = 0.032), and glyoxylate and dicarboxylate metabolism (*P* = 0.041) were enriched significantly (Figure 3D).

**TABLE 2:** Different metabolites in the pituitary gland between the CON and TR groups.

Name	VIP	FC	*P*-value
**Fatty acids and lipids**			
Oxoglutaric acid	1.689	37.176	0.009
Capric acid	1.831	19.337	0.009
Phosphorylcholine	1.591	0.564	0.047
**Amino acids and derivatives**			
L-serine	1.746	10.270	0.028
Aspartame	1.889	0.529	0.016
L-Kynurenine	1.438	0.406	0.047
N-Acetylhistidine	1.597	0.355	0.047
beta-Citryl-L-glutamate	1.845	0.205	0.028
L-glutamic acid	1.811	0.114	0.009
Oxidized glutathione	1.688	0.253	0.047
L-3-Phenyllactic acid	1.534	0.649	0.028
**Nucleosides and nucleotides**			
Inosine	1.354	17.484	0.009
Deoxyuridine	2.208	0.648	0.009
**Sugars**			
D-ribose	1.923	0.156	0.016
Galactose 1-phosphate	1.663	0.402	0.047
Glucaric acid	1.802	0.181	0.047
Sucrose	1.932	0.658	0.028
**Vitamin**			
Vitamin D3	1.908	10.042	0.028
**Others**			
Levamisole	2.012	19.656	0.009
1,3-Benzenediol	1.785	0.273	0.028
4-Pyridoxic acid	1.300	0.378	0.047
4-Quinolinecarboxylic acid	2.172	0.312	0.009
Dibutyl phthalate	1.690	0.179	0.047
Cyclopeptine	1.727	0.206	0.047

### 3.6. Undernutrition influenced ribosomal structure and biogenesis for translation and cytoskeleton

**FIGURE 4:**
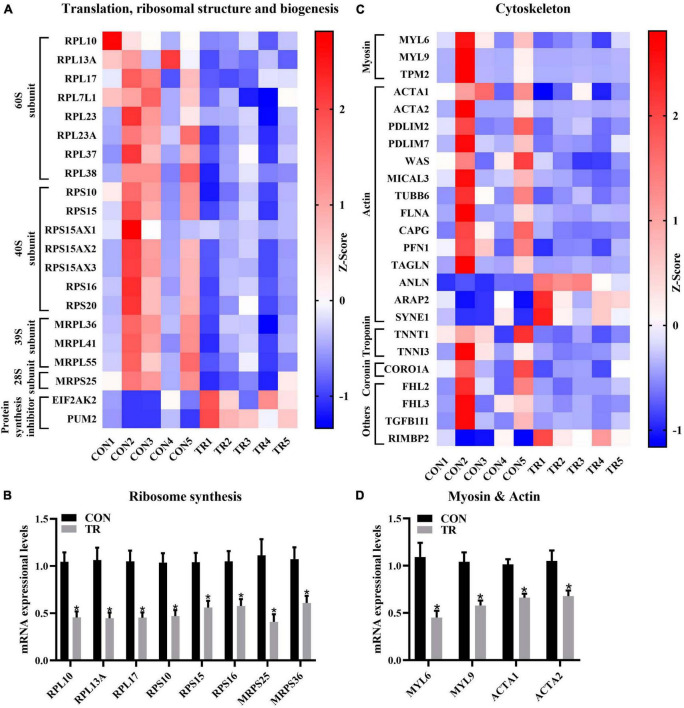
Undernutrition inhibited ribosomal structure and biogenesis and cytoskeleton of the pituitary gland. **(A)** Heatmap of the expressional levels of DEGs involved in translation, ribosomal structure, and biogenesis in the pituitary gland between the CON and TR groups. **(B)** The expression of genes related to ribosomes synthesis in quantitative real-time PCR. **(C)** Heatmap of the expressional levels of DEGs involved in cytoskeleton in the pituitary gland between the CON and TR groups. **(D)** The expression of genes related to myosin and actin in quantitative real-time PCR. Data were presented as the mean with SEM. DEGs were selected based on FDR < 0.05 and FC > 2 or <0.5 (*n* = 5 per group). The difference of gene expression in quantitative real-time PCR was identified by independent sample *t*-test (*n* = 10 per group), and asterisk indicated the significant difference (*P* < 0.05).

KOG functional classification of DEGs in the pituitary gland showed translation, ribosomal structure and biogenesis (J), and the cytoskeleton (Z) were enriched by DEGs. In the current study, DEGs related to the 60S subunit (RPL10, RPL13A, RPL17, RPL7L1, RPL23, RPL23A, RPL37, and RPL38), 40S subunit (RPS10, RPS15, RPS15AX1, RPS15AX2, RPS15AX3, RPS16, and RPS20), 39S subunit (MRPL36, MRPL41, and MRPL55), and 28S subunit (MRPS25) were all downregulated in the TR group compared to the CON group (Figure 4A). DEGs associated with inhibition of protein synthesis including EIF2AK2 and PUM2 were upregulated in the TR group (Figure 4A). RT-qPCR results also confirmed the lower mRNA expressional levels of genes related to ribosomes and mitoribosomes genesis, including RPL10 (*P* < 0.001), RPL13A (*P* < 0.001), RPL17 (*P* < 0.001), RPS10 (*P* < 0.001), RPS15 (*P* = 0.001), RPS16 (*P* = 0.002), MRPS25 (*P* = 0.002), and MRPS36 (*P* = 0.005) in the TR group (Figure 4B).

For the cytoskeleton, MYL6 and MYL9 encode myosin light chains while TPM2 participates in the synthesis of tropomyosin. They were all downregulated in the TR group compared to the CON group (Figure 4C). All of the DEGs related to actin, such as actin synthesis (ACTA1 and ACTA2), actin filament synthesis (PDLIM2, PDLIM7, and WAS), actin filament decomposition (MICAL3), and other actin forms including tubulin, filamin, capping actin, profilin, and transgelin (TUBB6, FLNA, CAPG, PFN1, and TAGLN), were downregulated in the TR group (Figure 4C), whereas DEGs involved in actin binding (ANLN) and actin cytoskeletal remodeling (ARAP2 and SYNE1) were upregulated in the TR group compared with the CON group. TNNT1 and TNNI3, which are involve in encoding troponin T and troponin I, were downregulated in the TR group (Figure 4C). CORO1A, which is associated with coronin synthesis, was also downregulated in the TR group. In addition, several DEGs related to signaling transmission and protein interaction including FHL2, FHL3, TGFB1I1, and RIMBP2 were also significantly changed in the TR group (Figure 4C). Moreover, RT-qPCR results also confirmed the lower mRNA expressional levels of genes linked to myosin and actin genesis for cytoskeleton including MYL6 (*P* = 0.001), MYL9 (*P* = 0.001), ACTA1 (*P* < 0.001), and ACAT2 (*P* = 0.009) in the TR group compared to the CON group (Figure 4D).

### 3.7. Undernutrition influenced oxidative phosphorylation and energy production and conversion

**FIGURE 5:**
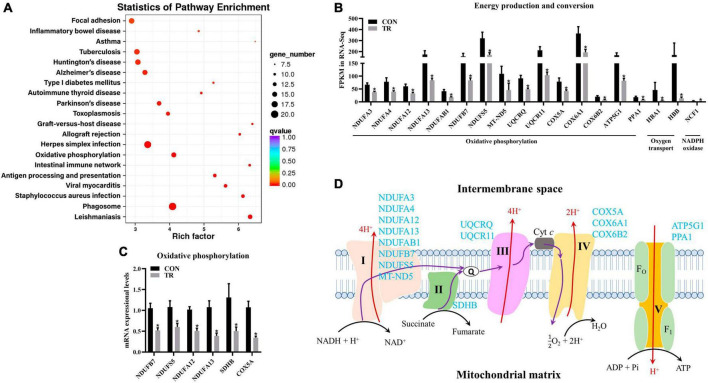
Undernutrition inhibited energy production and conversion of the pituitary gland. **(A)** KEGG pathway enrichment of DEGs in pituitary gland between the two groups. **(B)** The expression of genes related to energy production and conversion. **(C)** The expression of genes related to oxidative phosphorylation in quantitative real-time PCR. **(D)** Restricted feeding (TR) repressed oxidative phosphorylation in pituitary. Blue font indicates downregulated genes in the TR groups compared to the CON group. Data were presented as the mean with SEM. DEGs were selected based on FDR < 0.05 and FC > 2 or <0.5 (*n* = 5 per group). The difference of gene expression in quantitative real-time PCR was identified by independent sample *t*-test (*n* = 10 per group), and asterisk indicated the significant difference (*P* < 0.05).

As mentioned above, energy production and conversion were enriched in KOG functional classification, and oxidative phosphorylation was shown to be enriched in the KEGG pathway analysis by DEGs in the pituitary gland (Figure 5A). Oxidative phosphorylation is a biological pathway catalyzed by enzyme complex I, II, III, IV, and V to produce energy. Moreover, energy production and conversion and oxidative phosphorylation shared many DEGs, so these two terms were combined for analysis. In the current study, DEGs related to complex I (NDUFA3, NDUFA4, NDUFA12, NDUFA13, NDUFAB1, NDUFB7, NDUFS5, and MT-ND5), complex II (SDHB), complex III (UQCRQ and UQCR11), complex IV (COX5A, COX6A1, and COX6B2), and complex V (ATP5G1 and PPA1) in oxidative phosphorylation were all downregulated in the TR group compared to the CON group (Figures 5A, B). In addition, another three DEGs in the pathway of oxidative phosphorylation, including HBA1 and HBB, which are involved in oxygen transport, and NCF1, which encodes a subunit of neutrophil NADPH oxidase, were downregulated (Figure 5B). RT-qPCR results also confirmed the lower mRNA expressional levels of genes involved in oxidative phosphorylation including NDUFB7 (*P* = 0.001), NDUFS5 (*P* = 0.013), NDUFA12 (*P* < 0.001), NDUFA13 (*P* = 0.001), SDHB (*P* = 0.030), and COX5A (*P* < 0.001) in the TR group compared with the CON group (Figure 5C).

### 3.8. Undernutrition influenced phagosomes and immune responses

**FIGURE 6:**
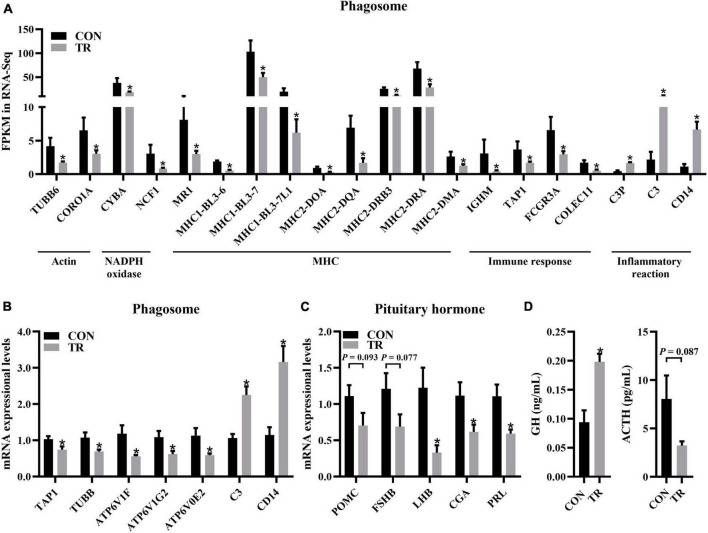
Undernutrition inhibited phagosome and hormone synthesis and secretion in the pituitary gland. **(A)** The expression of genes related to phagosome. **(B)** The expression of genes related to phagosome in quantitative real-time PCR. **(C)** The expression of genes related to pituitary hormone synthesis and secretion in quantitative real-time PCR. **(D)** Hormonal status of sheep between the CON and TR groups. Data were presented as the mean with SEM. DEGs were selected based on FDR < 0.05 and FC > 2 or <0.5 (*n* = 5 per group). The difference of gene expression in quantitative real-time PCR was identified by independent sample *t*-test (*n* = 10 per group), and asterisk indicated the significant difference (*P* < 0.05). *P < 0.05. GH, growth hormone; ACTH, adrenocorticotropic hormone.

In the KEGG pathway analysis, phagosomes were enriched significantly (*q* < 0.001) and contained most of the DEGs (Figure 5A). Some DEGs in the phagosome pathway involved in actin biosynthesis (TUBB6 and CORO1A) and NADPH oxidase (CYBA and NCF1) were downregulated and have been mentioned above (Figures 4C, 5B). DEGs involved in MHC1 (MR1, MHC1-BL3-6, MHC1-BL3-7, and MHC1-BL3-7L1) and MHC2 (MHC2-DOA, MHC2-DQA, MHC2-DRB3, MHC2-DRA, and MHC2-DMA) were all downregulated in the TR group (Figure 6A). These DEGs related to immune responses, including IGHM, TAP1, FCGR3A, and COLEC11, were all downregulated in the TR group (Figure 6A). Meanwhile, DEGs linked to inflammatory reactions, including C3P, C3, and CD14, were all upregulated in the TR group (Figure 6A). RT-qPCR results also confirmed the lower mRNA expressional levels of genes involved in oxidative phosphorylation, including TAP1 (*P* = 0.032), TUBB (*P* = 0.025), ATP6V1F (*P* = 0.015), ATP6V1G2 (*P* = 0.027), ATP6V0E2 (*P* = 0.021), C3 (*P* < 0.001), and CD14 (*P* = 0.001) in the TR group compared with the CON group (Figure 6B).

### 3.9. Undernutrition influenced some pituitary hormone synthesis and secretion

It is well known that the pituitary gland is involved in the synthesis and secretion of hormones. In this study, we performed RT-qPCR for genes involved in hormone synthesis and secretion to check whether this function of the pituitary gland was affected by undernutrition. The results showed that LHB (*P* = 0.007), CGA (*P* = 0.028), and PRL (*P* = 0.008) were all downregulated in the TR group compared with the CON group while POMC (*P* = 0.093) and FSHB (*P* = 0.077) showed no significant difference between the two groups (Figure 6C). Compared to the CON group, the TR group exhibited significantly higher levels of GH (*P* = 0.003). However, ACTH (*P* = 0.087) did not change significantly (Figure 6D), but there was a trend toward change.

## 4. Discussion

The pituitary gland is known as the “master gland” and regulates multiple target organs such as the adrenal gland, gonad, and thyroid gland (5). The most important function of the pituitary gland is to participate in the endocrine regulation of various hormones. Hormones in the body change strongly during pregnancy and puerperium (20). The pituitary gland plays a crucial role during normal pregnancy. In this study, we focused on the effect of undernutrition on the pituitary gland in a pregnant sheep model and related mechanisms. Nutritional status and dietary composition influence hormone synthesis, secretion, metabolism, and excretion (6). Long-term consumption of a high-energy diet can affect the secretion of pituitary-related hormones in ewes (21). Enhanced nutrition for early calves promotes the regulation of the hypothalamic-anterior pituitary-testicular axis (22), while malnutrition inhibits luteinizing hormone secretion in both rams and ewes (23, 24) and reduces gonadotropin-releasing hormone release from the pituitary gland (25). Although there has been extensive research about the effects of malnutrition on pituitary-related hormones, little is known about why malnutrition during late pregnancy affects the levels of pituitary-related hormones.

PCA and PLS-DA plots of total genes and total metabolites demonstrated that the general transcriptional and metabolic profiles of the pituitary gland were changed obviously upon undernutrition during late gestation. KOG functional classification of DEGs showed that substance transport and metabolism, including those of lipids, carbohydrates, and amino acids, were disrupted. In the current study, undernutrition enhanced lipid degradation and reduced lipid transport and synthesis in the pituitary gland, however, there is little literature about lipid metabolism in the pituitary gland upon undernutrition. Our previous study found increased fatty acid oxidation and decreased fatty acid synthesis in the liver of undernourished ewes (26). This probably implied that enhanced lipid degradation increased the energy supply and repressed lipid synthesis to decrease energy expenditure in the whole body upon undernutrition to adapt to the low energy conditions. Carbohydrate and amino acid metabolism are also important for the normal functions of the pituitary gland. It has been reported that amino acid supplementation can promote pregnancy development by changing pituitary function in the broodmare (27). However, little is known about the effects of undernutrition on pituitary carbohydrate and amino acid metabolism. In this study, DEG analysis showed that undernutrition disrupted carbohydrate transport and metabolism, enhanced protein metabolism and amino acid synthesis and transport, and repressed proteolysis and amino acid degradation in the pituitary gland of pregnant sheep.

In addition, metabolome analysis also clearly indicated the metabolic changes of amino acids and derivatives in the pituitary gland upon undernutrition. Aspartame is a dipeptide and is functionally related to an L-aspartic acid and a methyl L-phenylalaninate. Aspartic acid is the sole amino acid capable of oxidation within the brain and exhibits a prominent anti-fatigue effect (28). The level of glutamate in the brain is intricately linked to its energy provision (29). Oxidized glutathione plays a vital role in maintaining cellular redox homeostasis and functions as a signaling molecule in diverse cellular processes (30). Serine is synthesized in the forebrain and widely exists in the central nervous system (31). L-serine has been reported to play a neuroprotective role through a variety of biochemical and molecular mechanisms (32). In this study, undernutrition significantly reduced the levels of aspartame, glutamate, and oxidized glutathione and increased the level of L-serine in the pituitary gland, indicating the imbalanced metabolic homeostasis and energy metabolism in the pituitary gland of pregnant sheep.

It is well known that substance metabolism is usually accompanied by energy conversion and production. ATP synthesis in oxidative phosphorylation is mainly regulated by complex enzymes including NDUFs, UQCRs, COXs, and ATP synthases (33). In this study, several of DEGs involved in complex I, II, III, IV, and V and oxygen transport were all downregulated in the TR group, indicating that oxidative phosphorylation and energy conversion and production were severely inhibited in pituitary gland of undernourished ewes. So, undernutrition during late gestation affected some biological processes and energy conversion and production in the pituitary gland in a pregnant sheep.

The ribosome contains a 60S large subunit and a 40S small subunit and is the cellular site for protein synthesis (34); defects in ribosome production induces a wide variety of diseases (35–37). The ribosomes also presents in the mitochondria, called mitoribosomes, consist of a 39S subunit and a 28S subunit (38). All the DEGs involved in ribosome proteins in the hypothalamus of Hu-sheep were downregulated under heat stress, which reduced ribosome production and initiated apoptosis (39). In the present study, we found that DEGs related to the biosynthesis of ribosomes including cytoplasmic ribosomes and mitochondrial ribosomes in the pituitary gland were all severely inhibited upon undernutrition. It is also important to note that the production of ribosomes is an extremely energetically expensive cellular process (40). The inhibited ribosomal structure and biogenesis in the pituitary gland upon undernutrition is highly possibly caused by the severely repressed oxidative phosphorylation and energy production. Moreover, the activated EIF2AK2-coding protein can phosphorylate translation initiation factor EIF2S1 and inhibit protein synthesis (41). PUM2 plays an inhibitory role in translation and affects protein synthesis during cell differentiation (42, 43). The upregulated expression of EIF2AK2 and PUM2 also implied the inhibited protein biosynthesis, which might potentially link to the reduced ribosome and mitoribosome production. In addition, we also found cytoskeleton formation and myosin, actin, troponin, and coronin synthesis in the pituitary gland were inhibited upon undernutrition for pregnant ewes. There is a direct relationship between nutrient availability and porcine pituitary cell proliferation (44). In summary, undernutrition caused energy production and conversion barriers during late gestation of sheep inhibited ribosomal structure and cytoskeleton in the pituitary gland, which might further affect cell proliferation and apoptosis.

**FIGURE 7:**
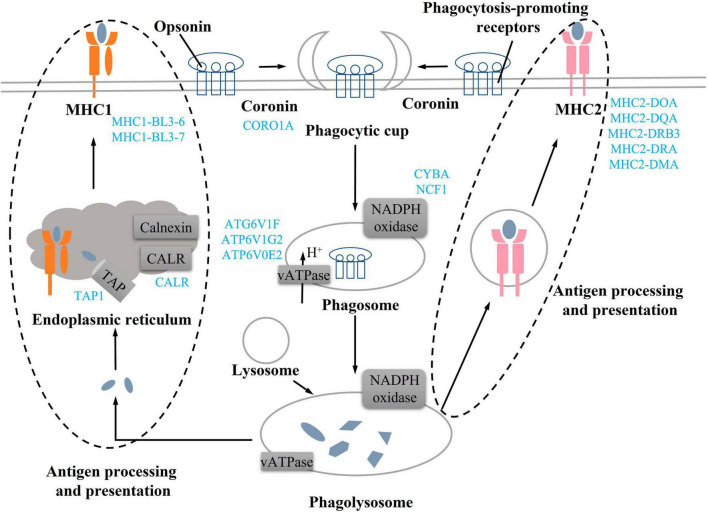
Undernutrition repressed phagocytosis process in the pituitary gland. Blue font indicates downregulated genes in the TR groups compared to the CON group.

Actin aggregation around the phagosome has been shown to contribute to the production of contractile forces (45–47). Coronin proteins are required for changes in cell morphology leading to migration and phagocytosis. (48). In addition, the deletion of coronin gene products reduces phagocytic function and cell chemotaxis (49). In this study, we found DEGs related to coronin, actin, and myosin synthesis were all downregulated, implying that these changes might affect phagocytic function. Phagosomes need to be reshaped through a series of independent events to mature (50) (Figure 7). Phagocytic cells first form phagocytic cups after binding to opsonin and phagocytosis-promoting receptors (51, 52), then, phagocytic cups recruit and activate the NADPH oxidase and v-ATPase to form the early phagosome (50, 52). The early phagosome gradually matures and combines with lysosomes to form phagocytic lysosomes, which can participate in immune response through antigen presentation (53, 54). Among them, v-ATPase mainly transports H + to the phagosome, changes its intramembrane pH, denatures the protein, and promotes protein degradation (55). NADPH oxidase consists of six subunits, including NOX2, p22phox, p47phox, p67phox, p40phox, and Rac, which need to be assembled together to produce the active complex (56). NADPH oxidase produces cellular reactive oxygen species, and the superoxide produced by the activated NADPH oxidase during the immune response can kill bacteria or fungi (57, 58). In this study, compared with the CON group, v-ATPase and NADPH oxidase-related DEGs in the process of phagolysosome formation were downregulated in the TR group, implying phagolysosome formation was inhibited. Both MHC1 and MHC2 can participate in antigen presentation (59, 60). MHC1 presents endogenous peptides to cytotoxic T cells via the classical pathway, while MHC2 presents antigens derived from extracellular proteins to T helper cells (61–63). Undernutrition has been reported to reduce immune defense and make individuals more susceptible to pathogens (64). In this study, it was found that DEGs related to MHC1 and MHC2 synthesis were all downregulated, implying that antigen presentation and processing in the phagocytosis was weakened in the pituitary gland of undernourished ewes. In addition, C3 is an acute phase reactant (65), which plays a central role in the activation of the complement system and participates in early inflammatory responses (66, 67) and immune regulation (68). C3 and C3P were all upregulated in the TR group, implying that inflammation might have occurred in the pituitary gland of undernourished ewes. Overall, undernutrition during late gestation impaired the immune system by influencing the phagosomes and the phagocytosis process and triggered an inflammatory reaction via complement cascades in the pituitary gland.

Both acute and chronic inflammation as well as dietary energy restriction can affect pituitary function and hormone secretion (69–71). The pituitary gland synthesizes and secretes four glycoprotein hormones, including chorionic gonadotropin, luteinizing hormone, follicle-stimulating hormone, and thyroid-stimulating hormone. These hormones are heterodimers formed by non-covalent binding of α and β subunits, with the α subunit being identical and the β chain being unique to each hormone. CGA encodes the α subunit, while FSHB and LHB encode the β subunits of follicle-stimulating hormone and luteinizing hormone, respectively. Our study found that genes related to luteinizing hormone and prolactin CGA, LHB, and PRL were downregulated upon undernutrition in pituitary gland, indicating that the synthesis of pituitary hormones might be affected. In sheep, undernutrition can inhibit luteinizing hormone secretion (23, 72) Nutritional disorders have been previously observed to disrupt the normal estrus cycle and modify luteinizing hormone secretion in pigs (73). Increased expression of FSHB in the pituitary gland is triggered by overnutrition in mid-gestation and late-gestation heifers (74). POMC is involved in the synthesis and secretion of pituitary adrenocorticotropic hormone, which has been shown to be downregulated by chronic feeding restriction in the pituitary gland of female lambs (75) and adult ewes (76). In the present study, the mRNA expressional levels of FSHB and POMC in the pituitary gland and the adrenocorticotropic hormone level in the blood all showed downward trends upon undernutrition in sheep, which were consistent with the changes mentioned above. In the current study, the growth hormone level was increased upon undernutrition in sheep, which was consistent with the elevated growth hormone levels in human (77), cattle (78), and chickens (79) under the condition of nutritional restriction. The rise in growth hormone concentration may indicate a transition in energy allocation from growth to maintenance (80). In short, undernutrition during late gestation affected genes involved in pituitary hormone synthesis and secretion and changed pituitary hormones in the blood.

**FIGURE 8:**
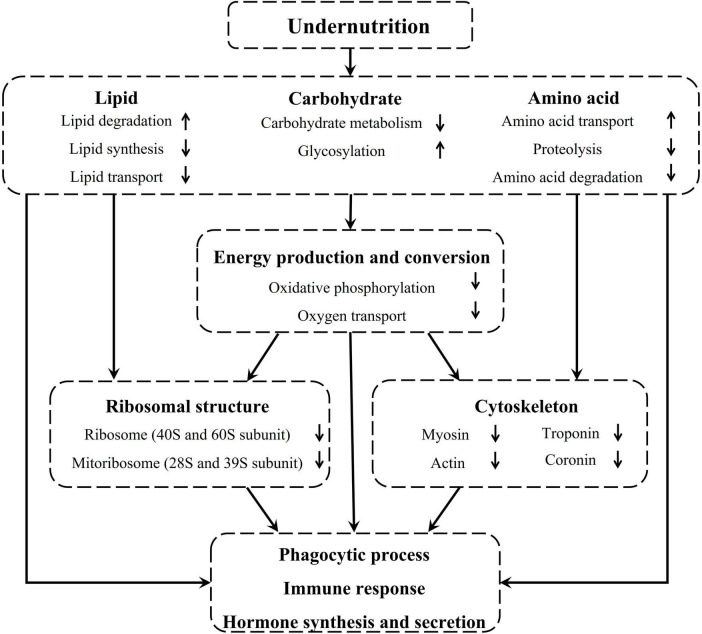
Undernutrition induced substance metabolism and energy production disorders affected the structure and function of the pituitary gland.

Taken together, undernutrition during late gestation disrupted the metabolism and transport of lipids, carbohydrates, and amino acids in the pituitary gland of pregnant sheep, which inhibited energy production and conversion and further repressed the regenesis of ribosomes and cytoskeleton. Finally, phagocytic process, immune function, and hormone synthesis and secretion were severely affected in the pituitary gland of undernourished ewes (Figure 8).

## 5. Conclusion

In conclusion, undernutrition influenced substances transport and metabolism in the pituitary gland of pregnant sheep, which inhibited energy production and cellular component synthesis, and consequently affected the function of the pituitary gland.

## Data availability statement

The original contributions presented in this study are publicly available. This data can be found here: National Center for Biotechnology Information (NCBI) Gene Expression Omnibus (GEO), https://www.ncbi.nlm.nih.gov/geo/, GSE220723.

## Ethics statement

The animal study was reviewed and approved by the Institutional Animal Care and Use Committee of Anhui Agricultural University (No. SYDW-P20190600601).

## Author contributions

YX and SM conceived and designed the study. SL, HL, and WZ conducted the research. SL, ZZ, and YX analyzed and interpreted the data. SL wrote the manuscript. YX and JC revised the manuscript. All authors read and approved the final version of the manuscript.

## Footnotes

1
http://www.hmdb.ca

2
http://www.lipidmaps.org

3
https://www.mzcloud.org

4
https://www.metaboanalyst.ca
