# Supplementary material for: Undernutrition-induced substance metabolism and energy production disorders affected the structure and function of the pituitary gland in a pregnant sheep model
Source: Front Nutr. 2023 Nov 15;10:1251936. doi: 10.3389/fnut.2023.1251936 (PMC10684748; doi:10.3389/fnut.2023.1251936)
Supplement: Supplementary file 1 [file Data_Sheet_1.docx]

# Supplementary data


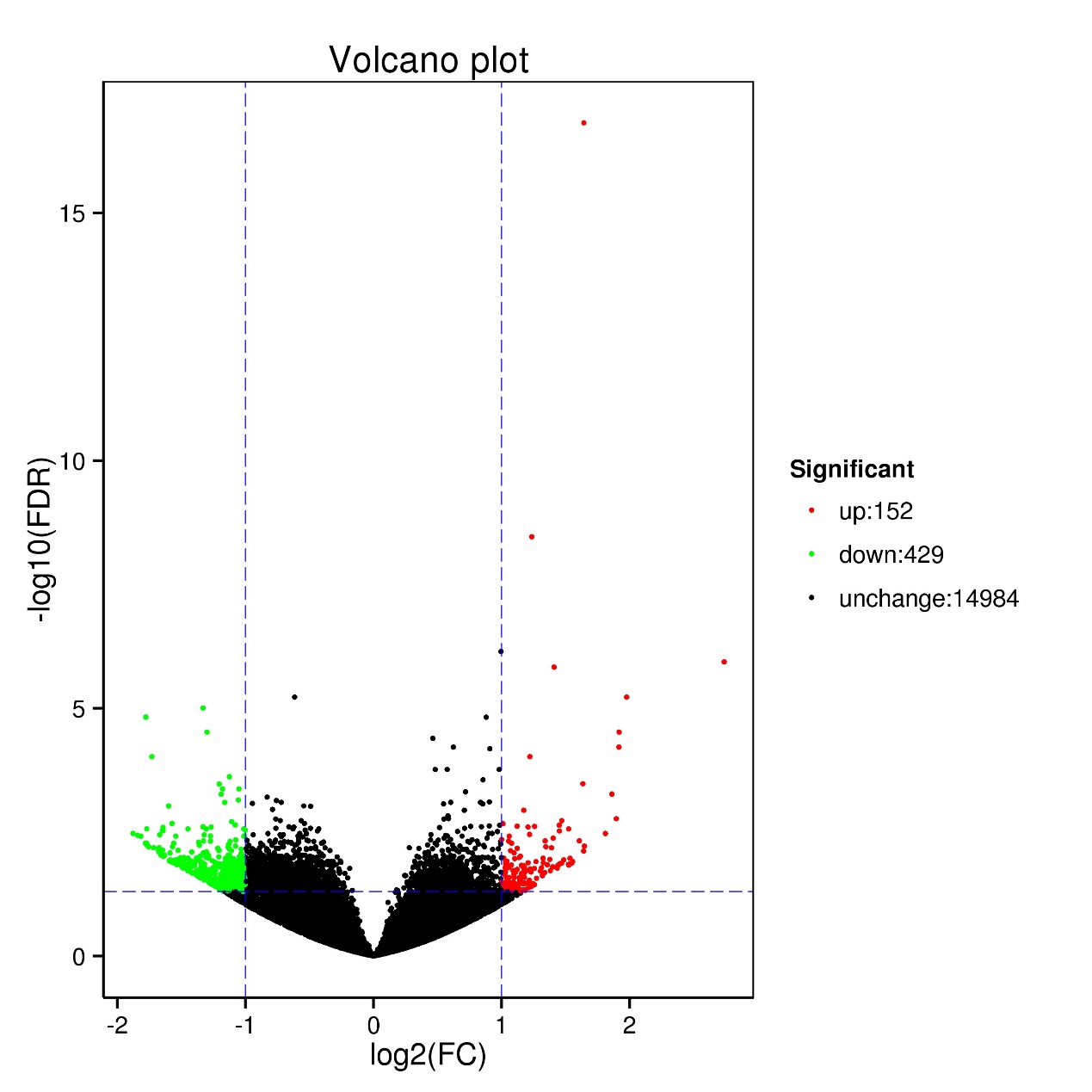


Supplementary file 1: Supplementary Fig. S1. Volcano plot shows DEGs in pituitary gland between the CON and TR groups. Red dots indicate upregulated genes while green dots indicate downregulated genes in the TR group with the criteria of FDR < 0.05 and FC > 2 or < 0.5.

Supplementary file 2: Supplementary Table S1 Ingredient composition and nutritional level of the total mixed ration.

| Items | Diets |
| --- | --- |
| Ingredient composition (% DM^1^) |  |
| Whole corn silage | 43.04 |
| Peanut straw | 31.07 |
| Maize | 17.79 |
| Bean pulp | 4.04 |
| Soybean meal | 2.70 |
| Premix^2^ | 1.36 |
| Nutrient composition |  |
| Metabolic energy (MJ/kg DM) | 11.25 |
| CP (% DM) | 12.60 |
| EE (% DM) | 3.20 |
| NDF (% DM) | 40.19 |
| ADF (% DM) | 34.20 |
| Crude ash (% DM) | 5.23 |
| Calcium (% DM) | 0.61 |
| Phosphorus (% DM) | 0.31 |

^1^DM = dry matter basis.

^2^The premix (per kg) contained: vitamin A 150,000 IU, vitamin D_3_ 25,000 IU, vitamin E 1000 mg, Zn 2548 mg, Cu 370 mg, Fe 1500 mg, Mn 637 mg, I 35 mg, Se 11 mg, lysine 1.5%.

Supplementary file 3: Supplementary Table S2 Gene primers used for quantitative real time-PCR.

Supplementary Table S2 Gene primers used for quantitative real time-PCR

| Gene symbol | Primer sequence | Accession number | Length (bp) |
| --- | --- | --- | --- |
| *SLC37A2* | F: GGTGATCCAGCCCCTCAATA | XM_012101850.4 | 153 |
|  | R: ATGCCACTGATGAACATGCC |  |  |
| *SLC35B4* | F: CTGTATCGGGTTCCAGTCGT | XM_004008076.5 | 170 |
|  | R: ATGAGGCTGACAAACTTGCG |  |  |
| *BCAT1* | F: TAAGAGCCTGGAAAGGTGGG | NM_001009444.1 | 244 |
|  | R: TGCCTCATCACTCCTGGAAG |  |  |
| *SLC38A1* | F: GTCTCACCAACAGCCACTTG | XM_012174434.4 | 243 |
|  | R: CCATGCAGCCTGTTTCCTTT |  |  |
| *SLC38A4* | F: GCGCATGTGGTGATGTTACT | XM_027967422.2 | 238 |
|  | R: CAGGGTGGCATACAAAAGCA |  |  |
| *RPL10* | F: CATGTGATTGAAGCCCTCCG | NM_001112820.1 | 177 |
|  | R: GGGACCACGATTAGGGATGT |  |  |
| *RPL13A* | F: GGATCCCACCACCCTATGAC | XM_012190698.3 | 166 |
|  | R: TCCTTTCGCTTCTCCTCCAG |  |  |
| *RPL17* | F: GGACTTACAGAGCTCACGGT | XM_027968632.2 | 171 |
|  | R: ATTCCCGGGCCATAAGTTTT |  |  |
| *RPS10* | F: CCAAGAAGAACCGGATTGCC | XM_015102661.3 | 179 |
|  | R: GGCAAACTGTTCCTTCACGT |  |  |
| *RPS15* | F: TGCGCGACATGATCATTCTG | XM_015096022.3 | 190 |
|  | R: GGGATGAAGCGGGAGGAAT |  |  |
| *RPS16* | F: TCATCAAGGTGAACGGACGA | XM_027978277.2 | 183 |
|  | R: AGGCTTTGGAGATGGACTGG |  |  |
| *MRPS25* | F: TCAAGGACTCGGTGAAGGTT | XM_015102172.3 | 179 |
|  | R: AGAATCCAGGTAGAAGCGCA |  |  |
| *MRPS36* | F: TCGTTCAGGTAGTCAAGCCA | XM_004016914.4 | 178 |
|  | R: GGTGGACCCTGATGCATC |  |  |
| *MYL6* | F: CTGTGGCCAAGAACAAGGAC | XM_004006589.4 | 212 |
|  | R: GCGGACGAGCTCTTCATAGT |  |  |
| *MYL9* | F: GTTTGGGGAGAAGCTGAACG | XM_027977163.2 | 170 |
|  | R: CCTCTCGGTACATCTCGTCC |  |  |
| *ACTA1* | F: CCCTGGAGAAGAGCTACGAG | XM_004021341.4 | 250 |
|  | R: GTGATCTCCTTCTGCATGCG |  |  |
| *ACTA2* | F: TCTGGACGTACAACTGGCAT | XM_004021341.4 | 212 |
|  | R: TTCTCCTTGATGTCCCGGAC |  |  |
| *NDUFB7* | F: TCCAGCAACGGGACTACTG | XM_004008468.5 | 157 |
|  | R: TCCCGCTCAAACTCCTTCAT |  |  |
| *NDUFS5* | F: CCGATGCCATGCTTTTGAGA | XM_004001824.4 | 199 |
|  | R: GTGAGGTGGAGGTGTGTACT |  |  |
| *NDUFA12* | F: CAGGGCAAATGATGTGAGGG | NM_001145188.1 | 159 |
|  | R: ATGCTTCCATCCACATCCCA |  |  |
| *NDUFA13* | F: TCGAAGGTGAAGCAGGACAT | XM_004008401.4 | 231 |
|  | R: GTCCTTTTCTGCCTGCAACA |  |  |
| *SDHB* | F: GCGCTCTGTATCGACTTGAC | XM_042244767.1 | 189 |
|  | R: CTTCTTGATTCGGGGTGCTG |  |  |
| *COX5A* | F: ATTGATGCTTGGGAGTTGCG | XM_027957175.2 | 164 |
|  | R: TGAGGTCCTGCTTTGTCCTT |  |  |
| *TAP1* | F: AACGTAACCCTCATGTCGGT | XM_004019165.5 | 206 |
|  | R: ATGGTGGACGTGTCATCTGT |  |  |
| *TUBB* | F: CAGTGTGGCAATCAGATCGG | XM_004018984.5 | 192 |
|  | R: GTCCATGGTTCCGGGTTCTA |  |  |
| *ATP6V1F* | F: AGACGAGGACACTGTGACTG | XM_004008049.5 | 238 |
|  | R: GATCTCCAGTACTGCCGGAA |  |  |
| *ATP6V1G2* | F: AAGGAGGAGGCACAAATGGA | XM_004018963.5 | 172 |
|  | R: GTTCACGGTTTCTCTGCTGG |  |  |
| *ATP6V0E2* | F: TCCCGGTCGTCATCTTCAC | XM_012177362.3 | 193 |
|  | R: GTCTCATTCTTCAGCTGGGG |  |  |
| *C3* | F: CAGACAAGGGGCACAAGTTC | XM_027969774.2 | 215 |
|  | R: GGGAGTCTCGATGCTGATGA |  |  |
| *CD14* | F: TCAATTTGTCGTTCGCTGGG | NM_001077209.2 | 197 |
|  | R: GGAGATCATCGGGTCGTCTT |  |  |
| *POMC* | F: GAGTATGGTCTGGTGGCAGA | XM_042245192.1 | 183 |
|  | R: GTGGGCGTTCTTGATGATGG |  |  |
| *FSHB* | F: CTATTGCTACACCCGGGACT | NM_001009798.1 | 150 |
|  | R: GTGGCTACTGGGTACGTGTA |  |  |
| *LHB* | F: TCACTTTCACCACCAGCATC | NM_001009380.1 | 245 |
|  | R: CAGGGGTTGGGTTCTGGG |  |  |
| *CGA* | F: CCAATTTATCAGTGCATGGGGT | NM_001009464.1 | 163 |
|  | R: TCTCCACTCTGACATTTCCCA |  |  |
| *PRL* | F: CTTCGAGACCTGTTTGACCG | NM_001009306.1 | 197 |
|  | R: AGGACTTCATGGTGGGTCTG |  |  |
| *PROP1* | F: TTCCAGAACCGCAGAGCTAA | NM_001009767.1 | 192 |
|  | R: GGAGGGCTGAGATGGAAGAG |  |  |
| *LHX3* | F: CACGACTATCACAGCCAAGC | XM_027966194.2 | 208 |
|  | R: GTTTCGGAAATACTGGCCCC |  |  |
| *HESX1* | F: TGCCGGAAGAAAGTGTTTGG | XM_012099865.4 | 244 |
|  | R: CGCCGATTTTGGAACCAGAT |  |  |
| *β-actin* | F: TCGTGATGGACTCTGGGGA | JN033788.1 | 160 |
|  | R: GCCGTGGTGGTGAAGCTGTA |  |  |
